# Debridement and corpectomy via single posterior approach to treat pyogenic spondylitis after vertebral augmentation

**DOI:** 10.1186/s12891-021-04478-0

**Published:** 2021-06-26

**Authors:** Shuai Zhang, Song Wang, Qing Wang, Jin Yang, Shuang Xu

**Affiliations:** Department of Orthopedics, The Affiliated Hospital of Southwest Medical University, NO. 25 Taiping Street, Sichuan 646000 Luzhou City, China

**Keywords:** Clinical efficacy, Debridement, Pyogenic spondylitis, Single posterior approach, Vertebral augmentation

## Abstract

**Background:**

Infection after vertebral augmentation (VA) often limits the daily activities of patients and even threatens their life. The operation may be one of the effective treatments if the patient suffers from intolerable severe pain, neurological deficits, and damage to spinal stability. This study aimed to investigate the clinical efficacy of the treatment of pyogenic spondylitis after vertebral augmentation (PSVA) with Single posterior debridement, vertebral body resection, and intervertebral bone graft fusion and internal fixation (sPVRIF).

**Methods:**

The study was performed on 19 patients with PSVA who underwent VA at 4 hospitals in the region between January 2010 and July 2020. Nineteen patients were included. Among them, 16 patients underwent sPVRIF to treat the PSVA.

**Results:**

A total of 2267 patients underwent VA at 4 hospitals in the region. Of the 19 patients with postoperative PSVA, suppurative spondylitis was misdiagnosed as an osteoporotic vertebral fracture(OVF) in 4 patients and they underwent VA. Besides osteoporosis, 18 patients had other comorbidities. The average interval between the first surgery and the diagnosis of PSVA was 96.4 days. Of the 19 patients, 16 received surgical treatment. The surgical time was 175.0±16.8 min, and the intraoperative blood loss was 465.6±166.0 mL. Pathogenic microorganisms were cultured in 12 patients.

**Conclusion:**

PSVA is a severe complication that can even threaten the life of the patients. sPVRIF may be one of the effective treatments if the patient suffers from intolerable severe pain, neurological deficits, and damage to spinal stability.

## Background

Vertebral augmentation (VA) has the advantages of immediate pain relief, minimal surgical trauma, and easy surgical techniques. Since its first clinical application by Galibert in 1984 [1], VA has gradually become one of the main methods for treating osteoporotic vertebral fractures(OVF). Despite the extensive development of VA, preventing multiple complications related to surgery is still a key issue that cannot be ignored in improving clinical efficacy. Previous studies reported that the incidence of pyogenic spondylitis after vertebral augmentation (PSVA) fluctuated at 0–1.6 %, which was low, but the consequences were severe and even threatened the lives of patients [2–4]. At present, most of the relevant studies on PSVA are case reports. These studies focused on the high-risk factors of postoperative infection, clinical manifestations after infection, and clinical diagnosis [5–16]. However, no in-depth research has been conducted on the surgical treatment of such patients. Antibiotics cannot form an effective antibacterial film around the infected lesion, and conventional anti-infective treatment measures are insufficient because polymethylmethacrylate (PMMA) is a nonbioactive artificial prosthesis. In addition, once such patients develop PSVA, they often experience unbearable severe pain, spinal deformity and loss of stability, and neurological deficits. Surgery inevitably becomes one of the main treatment methods for such patients. Park JW et al. [17] reported 11 patients with PSVA, 10 of which underwent surgery. The surgical procedure was thorough debridement of infected tissue and material, including PMMA following anterior column reconstruction via the anterior approach and percutaneous pedicle screw fixation. However, patients with OVFs are mostly older people with multiple comorbidities; they cannot bear the trauma caused by one-stage anterior and posterior surgery. Most spinal diseases can be treated via a single surgical approach with the familiarity of spinal anatomy and the highly developed surgical instruments related to spinal surgery. This study was novel in using single posterior debridement, vertebral body resection, and intervertebral bone graft fusion and internal fixation (sPVRIF) to treat PSVA so as to shorten the surgical time, reduce the surgical trauma, and avoid interference with the abdominal organs of the patient. The relevant surgical techniques, surgical experience, and preliminary results of the surgery are discussed in the following sections.

## Methods

All patients with postoperative spinal infections between January 2010 and July 2020, who primarily underwent VA, were examined after obtaining approval from the institutional review board of the hospital. Postoperative infection occurred in 9 patients (out of 1286 patients operated, with an infection rate of 0.7 %), and 10 were referred from the other 3 institutions for the treatment of infection (out of 981 patients operated, with an infection rate of 1.0 %). Finally, 19 patients were included in this study (Table 1).

**Table 1:** Demographics and comorbidities of patients and information on primary intervention

Cases	Level(s) of fracture	Comorbidities	Type of intervention	CRP (mg/L)	ESR (mm/h)	WBC (10^9/L)	VAS	Neu (%)	Biopsy	Level of infection/type of fracture
1	T9,L2	COPD,DM	KP	7.2	28	7.6	6	49	Negative	L2/OVCF
2	L3	HTN,IHD	VP	NA	NA	NA	NA	NA	NA	L3/OVCF
3	T10,T11,T12	HTN,CIS	KP	10.7	46	6.4	7	62	NA	T10/OVBF
4	L4	HF,Af	VP	8.3	19	6.7	5	63	Negative	L4/Kummell
5	L2	HTN,DM,UTI	KP	21.3	44	7.8	6	74	Chronic inflammation	L2/OVBF
6	L1,L2	DM,CIS	KP	17.6	36	8.2	6	68	NA	L2/Kummell
7	T11	Malnutrition,RI	KP	11.4	29	5.4	5	59	NA	T11/OVBF
8	T10,T11	Obesity,HTN	KP	13.6	33	5.9	4	62	NA	T10/OVCF
9	L3,L4	COPD,CIS	VP	NA	NA	NA	NA	NA	NA	L4/kummell
10	T8,T12	DM,Malnutrition	KP	8.3	16	6.2	6	63	Negative	T12/OVBF
11	L1	None	KP	27.9	67	4.6	5	41	Negative	L1/kummell
12	T12	SLE	KP	24.4	58	5.0	6	43	NA	T12/OVCF
13	L1,L2	PCa(chemotherapy)	VP	19.6	41	3.9	5	39	NA	L2/Kummell
14	T11	Obesity,CIS	VP	10.0	21	6.5	5	60	Negative	T11/OVCF
15	T10,L1	HTN	KP	8.1	18	7.1	6	67	Negative	L1/OVCF
16	T11,T12	UTI,DM	VP	20.4	39	8.5	6	79	NA	T12/kummell
17	L1	DM,HTN	VP	11.3	20	8.2	6	69	NA	L1/OVBF
18	L3,L4	COPD,RI	KP	8.3	17	7.1	4	63	NA	L4/OVCF
19	T12,L1,L2	Malnutrition, Decubitus	KP	7.0	42	8.0	6	70	NA	L2/OVBF

*CRP,C*-reactive protein, *ESR* erythrocyte sedimentation rate, *WBC *white blood count, *VAS* visual analog scale, *Neu* neutrophil ratio, *COPD* chronic obstructive pulmonary disease. *DM* diabetes mellitus, *KP* Kyphoplasty, *VP* vertebroplasty, *HTN* hypertension, *IHD* ischemic heart disease, *NA* not available, *CIS* cerebral ischemic stroke, *HF *heart failure, *Af* artrial fibrillation, *UTI* urinary tract infection, *RI* renal insufficiency, *SLE* systemic lupus erythematosus, *PCa* prostatic cancer, *OVCF* Osteoporosis vertebral body compression fracture, *OVBF* Osteoporosis vertebral body burst fracture

PMMA was used for all patients. Kyphoplasty was performed in 12 and vertebroplasty in 7 patients. The baseline data of the patients, such as sex, age, comorbidities, C-reactive protein (CRP) level, erythrocyte sedimentation rate (ESR), and visual analog scale (VAS) scores, were recorded before the first and the second surgeries. After the patient was diagnosed with PSVA, urine culture, sputum culture, and blood culture were routinely performed. Blood for blood culture should be drawn blood before using antibiotics besides drawing blood during chills. In addition, three blood samples from both sides of the human body are required for blood culture to increase the detection rate of pathogenic microorganisms. One of the 19 patients in this study gave up treatment, 2 chose conservative treatment, and 16 chose surgery, based on whether the patient had severe pain, neurological deficits, and loss of spinal stability, combined with the patient’s own desire for treatment.

**Fig. 1:**
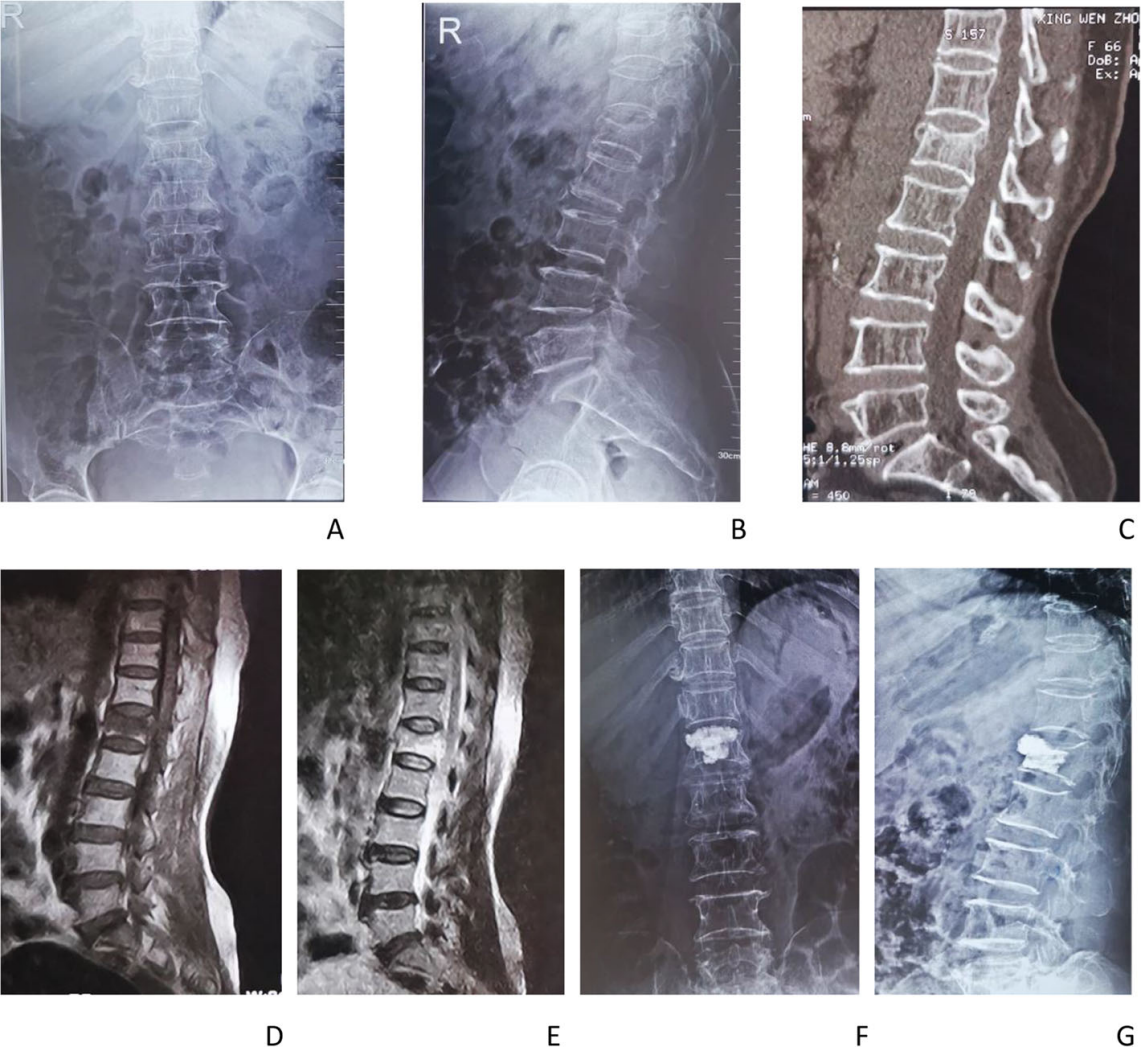
A patient in this study suffered an OVF of the L1 vertebra due to a lumbar sprain. **A–E** Imaging data before PKP surgery did not indicate vertebral infection. **F–G** Re-examination after PKP showed that the height of the vertebral body was partially restored, and the location of bone cement in the vertebral body was satisfactory

**Fig. 2:**
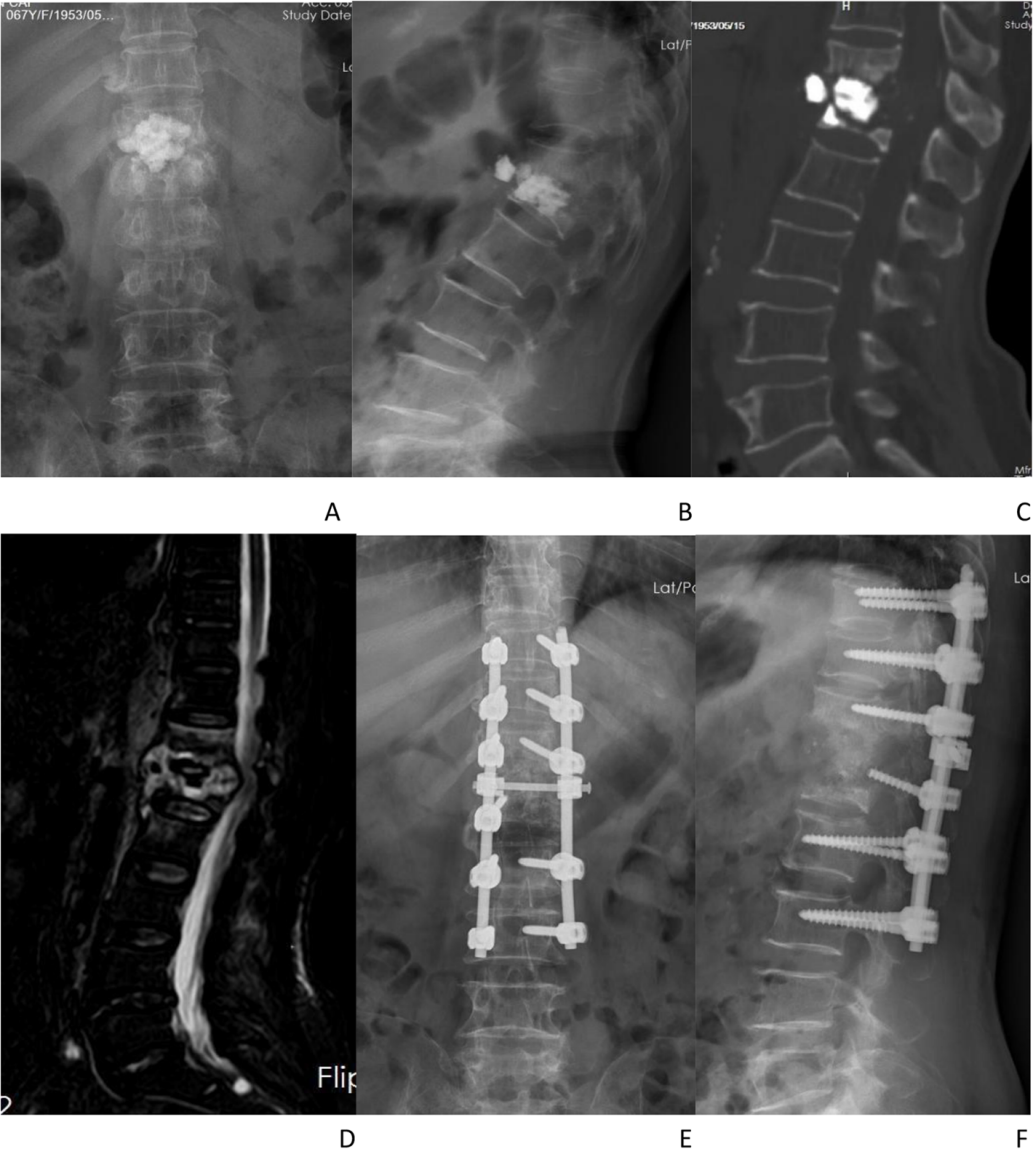
After 3 months of PKP surgery, the patient was hospitalized again with severe lower-back pain and paraplegia of both lower limbs. **A–D** Imaging examination revealed that the bone cement in the L1 vertebral body was displaced, the bone of the T12/L1 vertebral body was destroyed, the thoracolumbar segment formed a kyphotic deformity centered on T12/L1, the corresponding level of spinal canal stenosis, and the dural sac compression. **E–F** Single posterior decompression of lesions and bone cement removal, spinal canal decompression, intervertebral bone graft fusion, pedicle screw internal fixation surgery, and postoperative spinal sequence complete recovery

The brief procedure of the surgery was as follows. The patient’s information was checked. Tracheal intubation was performed under general anesthesia, and the patient was placed in a prone position, with a suspended abdomen. Before the surgery, the C arm was used to confirm the surgical site, and the surgical area was sterilized with 5 % iodophor solution three times. The skin and subcutaneous tissue were incised, and the bilateral paravertebral muscles were separated and exposed. The Wiltse approach was used to implant the pedicle screw of the intended fixed segment (the decision to implant a shorter pedicle screw into the destroyed vertebral body was made based on the extent of the destruction, and the basic principle was that the screw should not be close to the local lesion). The C-arm fluoroscopy pedicle screw was in a good position. One side was selected as the channel for lesion and PMMA removal, while a temporary connecting rod was placed on the other side. The articular joints, lamina, pedicle, and other structures of the destroyed vertebrae were exposed through the compartment of muscles between the longis and multifidus muscles. Piezosurgery was used to remove one side of the lamina, pedicle, facet joint, and other structures. The dural sac and corresponding nerve roots were protected, the destroyed vertebral body and PMMA were removed in piecemeal, and the necrotic tissue around the destroyed vertebral body was completely removed. The tissues at the junction of necrosis and normal tissues were selected for bacterial culture and pathological examination. The use of cages or metal implants to reconstruct bone defects caused by pyogenic spondylitis is still controversial. Cages or metal implants were found to hinder the healing of the infection; an increased rate of aseptic loosening has been reported in published studies [18–20]. If a local infection recurs in patients using cages or metal implants, the implants need to be removed, which may be fatal for elderly patients. In order to control the local infection better, we chose the autogenous iliac bone or rib to fill the bone defect according to the lesion location and reconstructed the spinal stability; then, bilateral connecting rods and transverse connectors were placed (Figs. 1 and 2). A plasma drainage tube was placed in the surgical area, and the surgical incision was closed. After the surgery, the patient was sent to the intensive care unit and transferred to the general ward after the condition was stable.

After the surgery, the surgical area was flushed through the plasma drainage tube using sterile normal saline for 7–10 days. If pathogenic microorganisms were detected before the surgery, antibiotics were selected based on the drug sensitivity test after the surgery and infused intravenously for 2 weeks and orally for 6 weeks. If no evidence of pathogenic microorganisms was found before the surgery, antibiotics were empirically injected with higher bone tissue distribution. After detecting pathogenic microorganisms, the antibiotics were adjusted according to the results of the drug sensitivity test. If pathogenic microorganisms could not be cultivated, empirical intravenous infusion of antibiotics with a higher bone tissue distribution was used for 2 weeks and oral administration for 6 weeks. The conservative treatment mainly involved the use of antibiotics, and the principles of antibiotic use were the same as that of surgical treatment.

### Statistical analysis

Statistical analysis was performed using the commercial software package SPSS 19.0 (SPSS, IL, USA). All results for continuous variables were presented as mean ± standard deviation, while those for categorical variables were expressed as *n*.

## Results

From January 2010 to July 2020, 2267 patients underwent VA at 4 hospitals in the region. Further, 19 had PSVA, and only 7 underwent biopsy simultaneously, of which 6 showed negative results in pathological examination, and 1 was detected with chronic low-virulence infection of the vertebral body. The patient’s medical history, inflammation indicators, and imaging examinations revealed that four were highly suspected of having suppurative spondylitis before undergoing VA. Among the 19 patients, 5 were men and 14 were women, with an average age of 73.0 (59–90) years. Besides osteoporosis, 18 patients had other comorbidities, among which hypertension and diabetes were the most common. Kyphoplasty was performed in 12 and vertebroplasty in 7 patients. Before VA, the CRP level, ESR, and other inflammatory indexes were completely normal in seven patients. Of the 19 vertebrae infected, 7 were osteoporosis vertebral body compression fracture, 6 were osteoporosis vertebral body burst fracture, and 6 were Kummell’s disease (Table 1).

The average time interval between VA and diagnosis of PSVA was 96.4 (14–373) days. According to Abdelrahm’s et al. [10] diagnostic criteria, 4 patients had an acute infection (within 1 month) and 15 had a chronic infection (more than 1 month). Further, 10 patients had fever or chills during the course of the disease. After the occurrence of PSVA, the CRP level, ESR, white blood cell (WBC) count, and neutrophil (Neu) count were significantly higher than the normal values in patients. The VAS score reached 7.6 (6–10) points. Among the 19 patients with PSVA, 7 developed neurological deficits (Table 2).

**Table 2:** Clinical, Laboratory, preoperative data of reoperation

Cases	Reoperation after infection(d)	Fever/Chill	Neurologic deficites	CRP (mg/L)	ESR (mm/h)	WBC (10^9/L)	VAS	Neu (%)
1	67	+	None	45.7	62	11.4	7	77
2	103	+	None	81.3	59	9.8	7	81
3	14	-	None	109.7	48	13.5	8	89
4	88	-	Paraparesis	35.6	109	10.9	9	82
5	97^a^	-	Radiculopathy	40.2	77	9.6	9	79
6	135	+	Paraparesis	18.9	68	11.1	7	80
7	373	+	None	22.7	60	9.0	6	69
8	26^b^	-	Paraparesis	173.7	114	15.2	7	91
9	54	-	None	40.9	95	8.3	9	83
10	61	+	None	73.8	87	10.5	8	74
11	108	-	None	36.9	124	11.7	7	79
12	157	+	Radiculopathy	18.7	108	9.5	6	71
13	19	-	None	142.3	77	14.6	7	88
14	182^a^	-	Paraparesis	40.6	84	10.8	9	82
15	71	+	None	87.7	79	12.3	8	87
16	114	-	None	39.0	56	10.4	7	70
17	93	+	Paraparesis	45.5	38	7.9	6	69
18	21	+	None	109.0	103	10.8	10	78
19	49	+	None	92.5	81	14.1	8	89

*CRP *C*-*reactive protein, *ESR* erythrocyte sedimentation rate, *WBC *white blood count, *VAS* visual analog scale, *Neu* neutrophil ratio

^a^Conservative treatment

^b^Abandoning therapy

Among the 19 patients with PSVA, 14 completed the follow-up, with an average follow-up time of 14.3 (33 ± 3.5) months. Sixteen patients finally chose surgery due to unbearable severe pain, neurological deficits, and spinal instability. The surgical method was sPVRIF. All patients had a smooth surgery. The surgical time was 175.0 ± 16.8 (155–210) min, and the intraoperative blood loss was 465.6 ± 166.0 (300–900) mL. After surgical treatment, one patient died of refractory septic shock, one died of prostate cancer, and two still needed to live in a wheelchair. The daily activities of the remaining patients significantly improved after the surgery. The VAS score at the last follow-up was 1.8 (1–3) points. Among the patients treated conservatively, one died of refractory septic shock and one needed to use a walker for assistance in completing daily activities. The patient who gave up treatment eventually died. Among the 14 patients who completed the final follow-up, spontaneous fusion occurred in one patient with conservative treatment. Of the 13 patients who underwent an operation, 3 had a partial interbody fusion, and the follow-up time was less than 8 months. The remaining 10 had a complete fusion. A total of 12 of the 19 patients showed evidence of pathogenic microorganisms, among which the most common pathogenic bacteria were *Enterococcus faecalis* and *Streptococcus* (Table 3).

**Table 3:** Operative and postoperative data

Cases	Treatment method	Operative time(min)	Blood loss(ml)	Causative	Follow-up(month)	Outcome	VAS follow-up	Bony fusion
1	Operation	165	500	No organism	9.2	Normal	2	Fusion
2	Operation	190	400	Enterococcus faecalis	5.3	Normal	1	Partial fusion
3	Operation	185	350	Non-tuberculous mycobacterium	Died	NA	NA	NA
4	Operation	210	450	Micromonomonas	17.5	Wheelchair	2	Fusion
5	Conservative treatment	NA	NA	NA	Died	NA	NA	NA
6	Operation	180	400	No organism	23.5	Use a walker	3	Fusion
7	Operation	170	300	MRSE	8.5	Normal	2	Fusion
8	None	NA	NA	NA	Died	NA	NA	NA
9	Operation	160	350	Tread fungus	33.5	Use a walker	1	Fusion
10	Operation	160	300	S.albus	23.0	Normal	1	Fusion
11	Operation	210	650	Streptococcus	12.0	Normal	2	Fusion
12	Operation	155	300	No organism	7.5	Wheelchair	2	Partial fusion
13	Operation	165	400	S.aureus	15.5	Died(cancer)	NA	Fusion
14	Conservative treatment	NA	NA	NA	24.0	Use a walker	3	Spontaneous fusion
15	Operation	180	900	Enterococcus faecalis	3.0	Normal	1	Partial fusion
16	Operation	160	700	S.aureus	NA	NA	NA	NA
17	Operation	165	400	No organism	8.0	Normal	2	Fusion
18	Operation	170	500	Streptococcus	NA	NA	NA	NA
19	Operation	175	550	Escherichia coli	10.0	Normal	1	Fusion

*VAS* visual analog scale, *NA* not available, *MRSE* methicillin resistant Staphylococcus epidermidis, *S.albus* Staphylococcus albus, *S.aureus* Staphylococcus aureus

## Discussion

VA can immediately rebuild the stability of the spine and effectively relieve pain through the insertion and support between the PMMA and the fractured end of the vertebral body. However, PMMA leakage, refracture, PMMA toxicity, and PSVA are still important complications that restrict the wide clinical application of VA. The incidence of PSVA is low, but the consequences are grave, even threatening patients’ lives. Since Yu et al. [5]first reported the PSVA, different scholars have summarized the characteristics, clinical manifestations, diagnostic methods, and treatment principles of PSVA. The PMMA currently used in clinic has no biological activity, and the polymerization of PMMA monomer leads to an increase in temperature inside the vertebral body. The bone tissue that is burnt and necrotic often forms an isolation zone between the PMMA and the normal bone tissue. The aforementioned factors are all important reasons why it is challenging to cure PSVA using conservative treatment. Patients with PSVA often suffer from uncomfortable severe pain, local kyphosis, and neurological deficits. Hence, it is difficult to achieve satisfactory results with antibiotics alone. Surgical treatment has become an indispensable choice for such patients. However, in-depth and systematic research on the surgical method of such patients lacks due to the small sample size. The scholars in the past often recommended the use of anterior debridement combined with posterior internal fixation for the treatment of PSVA to completely remove the lesions, pus, and PMMA [3, 6, 10, 17, 21–23]. However, such patients often have multiple diseases due to their advanced age, and multiple-organ dysfunction throughout the body can hardly tolerate such large surgical trauma. Since the 21st century, most spinal diseases could be treated via a single approach with an in-depth understanding of the pathophysiological mechanism of spinal diseases, anatomical structure of the spinal column, highly developed spinal surgical instruments, and increasing proficiency of surgical techniques. This study was performed on 19 patients with PSVA, which is the largest sample used so far. All surgical patients in this study were treated with sPVRIF. The surgical time was 175.0 ± 16.8 (155–210) min and the intraoperative blood loss was 465.6 ± 166.0 (300–900) mL, which were significantly lower than those reported in previous studies. This was mainly due to the simultaneous completion of lesion removal and internal fixation via a single surgical approach. In addition, piezosurgery is vital in the resection of the vertebral body and PMMA. Among the 16 patients with PSVA, 14 completed the last follow-up. The daily activities of the other patients significantly improved, except for two patients who still needed to be in a wheelchair after the surgery. Only one patient died of postoperative refractory septic shock, and the mortality rate was significantly lower than that reported by Abdelrahman. Besides improving the surgical method, the following improvements were made during the perioperative period: (1) Patients’ CRP, ESR, Neu, and other inflammatory indicators were dynamically observed besides following the basic principles of antibiotic use. The types of antibiotics used to avoid bacterial imbalance and double infections were dynamically adjusted based on the results of the drug sensitivity test. (2) The stability of the patient’s internal environment was maintained, and the nutritional status was improved. The plasma protein level of such patients was maintained at more than 35 g/L, and continuous maintenance of the hemoglobin level of more than 100 g/L was essential to enhance the patient’s disease resistance. (3) The surgical area for each patient was routinely flushed for 7–10 days after the surgery to reduce the concentration of local pathogens and inflammatory mediators. (4) The patients’ oral and perineal care was strengthened to prevent urinary tract and lung infections.

Previous studies [10, 12, 16, 17]reported that the incidence of PSVA was 0–1.6 %, and the incidence in this study was 0.83 %. Although the incidence was low, it resulted in catastrophic consequences to patients. Previous scholars [24] suggested the use of bone cement mixed with tobramycin for VA, while some recommended the use of perioperative intravenous prophylactic antibiotics to prevent the occurrence of such complications in high-risk patients. However, the clinical efficacy of the aforementioned methods still requires large-sample prospective comparative studies for validation. In this study, neither cement-loaded antibiotics nor systemic perioperative prophylactic intravenous antibiotics were used; instead, a single intraoperative prophylactic dose of a first-generation cephalosporin was used. The experience was as follows: (1) If the levels of inflammation indicators were elevated before the surgery, pulmonary infection and urinary tract infection needed to be carefully checked. If a clear infection of other parts was detected, it was recommended to perform VA 2 weeks after the infection was cured. (2) If infectious diseases of the vertebral body could not be ruled out, it was recommended to give priority to conservative treatment after 2 weeks and re-examine the magnetic resonance imaging and computed tomography of the fractured vertebral body. If it was an infectious disease, the progress of vertebral body disease was often found at this time. During conservative treatment, a biopsy of the destroyed vertebral body was performed. (3) Radionuclide bone scintigraphy can not only detect acute vertebral fractures early but also has unique advantages in differentiating spinal infectious diseases, tumors, and fractures [25, 26]. Therefore, we recommend routine radionuclide bone scintigraphy in patients with suspected OVF.

Obtaining etiological evidence is key to the treatment of infectious diseases. Patients with PSVA often start using antibiotics before obtaining pathogenic evidence, and it is generally difficult to cultivate pathogenic microorganisms. In addition, Vats HS et al. [13] reported that polymerase chain reaction (PCR) increased the detection rate of pathogenic microorganisms. Elderly people have long-term oral usage of multiple drugs to treat other basic diseases and lack personal hygiene and health protection knowledge, leading to significant changes in the bacterial spectrum of infectious diseases. Hence, routine urine culture, sputum culture, and blood culture are recommended. Blood for blood culture should be withdrawn before using antibiotics and during chills. In addition, three blood samples from both sides of the human body for blood culture are required to increase the detection rate of pathogenic microorganisms. While searching for pathogenic evidence, attention should be paid to rare pathogenic microorganisms, such as mycobacteria, fungi, and anaerobic bacteria.

This study was novel in reporting the clinical efficacy of sPVRIF in the treatment of PSVA. However, it had some limitations. This study was a single-center retrospective study and lacked comparative findings. Also, the follow-up time of this study was short.

## Conclusions

PSVA after VA is a severe complication and can even threaten the lives of patients. The possibility of vertebral infection should be ruled out before VA. Once diagnosed with suppurative spondylitis, pathogenic microorganisms should be detected through blood culture, bacterial culture of necrotic tissue at the lesion site, and PCR. The use of sPVRIF combined with the standardized antibiotic application is one of the effective treatment methods if the patient has intolerable severe pain, neurological deficits, and spinal stability damage.

## Data Availability

Data will be available upon request to the corresponding author Song Wang.

## Abbreviations

CRP: C-reactive protein
ESR: Erythrocyte sedimentation rate
Neu: Neutrophil
OVFs: Osteoporotic vertebral fractures
PCR: Polymerase chain reaction
PMMA: Polymethylmethacrylate
PSVA: Pyogenic spondylitis after vertebral augmentation
sPVRIF: Single posterior debridement, vertebral body resection, and intervertebral bone graft fusion and internal fixation
VA: Vertebral augmentation
VAS: Visual analog scale
WBC: White blood count
